# The Gut Microbiota–Epigenome Axis in Bariatric Surgery: Mechanistic Insights and Therapeutic Perspectives

**DOI:** 10.3390/metabo16100755

**Published:** 2026-10-09

**Authors:** Mohammed Y. Ezzi

**Affiliations:** Department of Surgery, College of Medicine, Jazan University, Jazan 45142, Saudi Arabia; mezzi@jazanu.edu.sa

**Keywords:** bariatric surgery, gut microbiota, epigenetics, microbial metabolites

## Abstract

The global prevalence of obesity has reached an alarming level, placing a significant burden on public health systems and individual well-being. Bariatric surgeries (BSs) result in greater and more sustained weight reduction than emerging medications such as glucagon-like peptide-1 (GLP-1). Despite the proven effectiveness of BS, considerable inter-individual variability in postoperative outcomes persists, with differences in weight loss and remission of obesity-related comorbidities among patients. Gut microbiota gene richness has been shown to change following BS. Importantly, the influence of the gut microbiota extends beyond conventional metabolic pathways to include interactions with the host epigenome. Although growing evidence suggests that microbiota-derived metabolites can influence epigenetic processes, whether inter-individual differences in gut microbiota composition contribute to variability in postoperative weight loss and metabolic outcomes following BS remains largely unknown. The gut microbiota–epigenome axis represents a potential molecular interface between postoperative gastrointestinal remodeling and metabolic adaptation. Surgical remodeling of the gastrointestinal tract reshapes the gut microbiota and its metabolites, including short-chain fatty acids and B vitamins, which may regulate host gene expression through histone deacetylase inhibition, DNA methylation, and modulation of systemic inflammation. Current evidence suggests that postoperative probiotic supplementation may improve glucose homeostasis, lipid profiles, and vitamin B12 and D status while having little or no additional effect on postoperative weight loss. Future research should address the limitations of existing studies by conducting adequately powered, high-quality randomized controlled trials integrating multi-omics technologies and precision medicine approaches to identify microbiome-based therapeutic targets, including postbiotic interventions. Further elucidation of this axis may support the development of precision bariatric strategies, although the clinical utility of microbiome- and epigenome-targeted interventions remains to be established.

## 1. Introduction

The global prevalence of obesity has reached an alarming level, placing a significant burden on public health systems and individual well-being. Obesity is more than just a cosmetic issue; it is a complex metabolic disease that leads to a number of serious comorbidities, including metabolic dysfunction-associated steatohepatitis (MASH), cardiovascular diseases, type 2 diabetes (T2DM), and certain cancers. More than 1.3 billion people globally are expected to develop diabetes by 2050, mainly driven by obesity. Moreover, the global impact of obesity-related complications is expected to intensify, with cardiovascular event rates expected to double within the next ten years, and obesity-associated cancers are anticipated to rise as well [1,2,3,4].

The Middle East and North Africa region has witnessed a substantial and rapid increase in obesity prevalence, with some countries reporting exceptionally high rates of obesity among adults [2]. The economic burden attributable to obesity-related healthcare expenses and diminished productivity reached more than 340 billion dollars in 2023 [4].

For many people dealing with severe obesity, the traditional weight reduction strategies and medications just do not deliver long-term success. Emerging pharmaceuticals such as glucagon-like peptide-1 (GLP-1) receptor agonists represent a significant therapeutic advance in the medical management of obesity and T2DM. Nevertheless, they have yet to demonstrate outcomes comparable in magnitude and durability to those achieved through metabolic and bariatric surgery (BS). Additionally, weight regain is frequent when treatment is discontinued, and the extent of weight loss is generally more modest. Bariatric surgeries, on the other hand, consistently result in a greater and more sustained weight reduction that can be maintained over many years. Beyond the weight loss itself, these surgeries trigger beneficial metabolic changes that can significantly improve overall health and quality of life [5]. Moreover, they lead to dramatic improvement and resolution of serious obesity-related comorbidities, including T2DM, heart disease, high blood pressure, and several other comorbidities that often accompany excess weight. Importantly, modern BS has a good safety record. Surprisingly, its risk profile is comparable to that of some of the safest and most routinely performed operations in the United States, such as cholecystectomy, appendectomy, and knee replacement [6,7,8].

Despite the proven effectiveness of BS, the results remain subject to significant variability in how individual patients respond to the procedure. Although most patients experience significant weight reduction and resolution of their comorbidities over years, a considerable subgroup experiences less favorable outcomes such as insufficient weight loss, significant weight gain over time, or only partial improvement in obesity-linked illnesses like T2DM. This wide variability in outcomes has puzzled researchers for years. Clearly, this heterogeneity underscores the complexity of obesity pathophysiology and suggests that factors beyond anatomical alterations contribute to the long-term success or failure of BS. These factors may include demographic characteristics, the ability to maintain necessary lifestyle changes after surgery, and psychological and behavioral elements. Understanding these contributing factors is essential to establish positive long-term outcomes following BS [9,10,11].

Importantly, emerging evidence points to a novel critical contributor that helps explain much of this variability: the gut microbiota. These trillions of microbes that inhabit the gastrointestinal tract do far more than just help digest food. They act like an internal control center that influences inflammation levels, hormone signals, energy use, and how our bodies adapt after surgery. Additionally, this dynamic gut ecosystem actively helps manage nutrient breakdown, supports immune function, and produces key metabolites that influence homeostasis [12,13].

In people with obesity and related conditions, the gut microbiome is often imbalanced, a state called dysbiosis. Additionally, a low microbial gene richness (MGR) was observed in them and was associated with metabolic alterations, including low-grade inflammation, insulin resistance, and adipocyte hypertrophy [14,15].

Importantly, the influence of the gut microbiota extends beyond conventional metabolic pathways to include interactions with the host epigenome, the regulatory network governing gene expression without altering the underlying DNA sequence. This raises the possibility that baseline gut microbiota composition may contribute to inter-individual variability in postoperative weight loss and metabolic outcomes following bariatric surgery [12]. The gut microbiota–epigenome axis therefore represents a plausible mechanistic framework through which microbial signals and metabolites may interact with host epigenetic processes. However, associations between microbiome changes and metabolic or epigenetic outcomes do not by themselves establish causality, particularly because bariatric surgery simultaneously alters gastrointestinal anatomy, nutrient flow, caloric intake, bile acid metabolism, body weight, and medication exposure. Therefore, microbiome-associated changes should be interpreted as potential contributors to postoperative metabolic and epigenetic remodeling rather than as established causal determinants.

The gut microbiota–epigenome axis represents a molecular interface through which microbial signals, particularly microbiota-derived metabolites, induce stable yet reversible changes in host gene expression via epigenetic mechanisms. By regulating inflammatory responses, metabolic pathways, and other physiological processes, this axis provides a novel framework for understanding how the altered gastrointestinal environment following BS may durably reprogram host biology. Consequently, it offers a plausible mechanistic explanation for both the remarkable metabolic benefits of BS and the substantial inter-individual variability in postoperative outcomes [16].

This narrative review aims to provide a comprehensive synthesis of the current understanding of the gut microbiota–epigenome axis in BS. We will first delineate the characteristic changes in gut microbial composition and function following different types of bariatric procedures. Subsequently, we will explore the specific mechanistic insights into how metabolites produced by these microbes, such as bile acids, short-chain fatty acids, and methyl donors, interact with the host’s epigenetic mechanisms, including effects on histone modifications, DNA methylation, and the regulation of non-coding RNAs, both directly and indirectly. Finally, we will highlight the therapeutic perspectives emerging from this research, including the potential for prebiotics, probiotics, fecal microbiota transplantation, synbiotics, and other future microbiome-targeted interventions to optimize surgical success and enhance long-term metabolic health in patients undergoing BS.

## 2. Key Terms

- Gut Microbiome: The collective genomes and functional genetic repertoire of the microorganisms inhabiting the human gastrointestinal tract. These microorganisms, including bacteria, archaea, fungi, viruses, and other eukaryotic microbes, form a dynamic ecosystem that influences host physiology in health and disease and is shaped by diet, genetics, medications, and surgical interventions [17].
- Epigenetics: The study of heritable and reversible changes in gene expression that occur without alterations in the underlying DNA sequence, primarily through DNA methylation, histone modifications, and non-coding RNAs [18].
- Short-Chain Fatty Acids (SCFAs): Organic acids that contain one to six carbon atoms. These acids include acetate, propionate, and butyrate, which are generated through bacterial fermentation of non-digestible dietary fiber by the gut microbiome [19].
- Bile Acids (BAs): Steroid molecules synthesized from cholesterol in the liver that facilitate lipid digestion and absorption. Primary bile acids are converted by the gut microbiota into secondary bile acids, which function as signaling molecules through receptors including the farnesoid X receptor (FXR) and Takeda G protein-coupled receptor 5 (TGR5), thereby regulating glucose metabolism, lipid homeostasis, inflammation, and energy expenditure [20,21,22,23,24].
- Microbiota–Epigenome Axis: A bidirectional communication pathway through which gut microbial metabolites and host-derived signals interact with epigenetic mechanisms to regulate gene expression, metabolism, immune function, and inflammatory responses [16].
- One-Carbon Metabolism: A metabolic pathway involving the transport of one-carbon units (such as methyl groups). The process supports the synthesis of S-adenosylmethionine (SAM), which serves as the universal methyl donor [25,26,27,28,29,30,31,32].
- DNA Methylation: A process that includes the addition of a methyl group (-CH3) to the 5′ carbon of a cytosine residue, typically within a CpG dinucleotide. This process is catalyzed by DNA methyltransferases (DNMTs) [33].
- Histone Modification: Post-translational chemical modifications of histone proteins, including acetylation, methylation, phosphorylation, ubiquitination, and crotonylation, that alter chromatin structure and regulate gene transcription [33].
- Metabolic Memory: The persistent biological effects of previous metabolic states, such as obesity or chronic hyperglycemia, that are maintained through stable epigenetic modifications despite subsequent metabolic improvement [12,34,35].

## 3. Gut Microbiome Before and After Bariatric Surgery

The human gut harbors a wide variety of microorganisms dominated by six main bacterial phyla: Firmicutes, Bacteroidetes, Verrucomicrobia, Actinobacteria, Euryarchaeota, and Proteobacteria. Together, Firmicutes and Bacteroidetes represent more than 90% of the total microbial population. Because of their dominance, they have received the most scientific attention. Interestingly, an elevated Firmicutes-to-Bacteroidetes ratio and reduced microbial variety have been found in obesity and have been associated with low-grade systemic inflammation.

BS helps reshape the gut microbiome. Both SG and RYGB typically increase Bacteroidetes and decrease Firmicutes, thereby lowering the Firmicutes/Bacteroidetes ratio toward a healthier balance. RYGB tends to have a more pronounced impact, including increasing Bacteroides, Proteobacteria, Veillonella, Escherichia, Streptococcus, and Slackia and decreasing Blautia. SG more prominently boosts Blautia, Eubacterium, Lactobacillus, and Haemophilus. Studies on Blautia showed that certain strains act as natural anti-hyperlipidemic agents by targeting lipid-modulating pathways and suppressing cellular fat accumulation. Additionally, restoration of microbiota diversity is often considered a shift toward a more metabolically healthy ecosystem [31,36].

The changes in microbiota composition are caused by gastric pH alteration, changes in nutrient availability and redistribution, modified intestinal transit times, and a bile acid enterohepatic circulation reconfiguration. These changes begin within weeks and often persist for months to years, contributing to sustained weight loss, improved metabolic health, and reduced appetite beyond the mechanical effects of the procedures. Overall, these taxonomic rearrangements favor microbes that strengthen the gut barrier and dampen inflammation [37,38].

## 4. Microbial Metabolites as Epigenetic Mediators After BS

Epigenetic regulation in the host is influenced by metabolites produced by the gut microbiota. These bioactive compounds can be produced or modified by microbial enzymes. They can cross the gut epithelium, enter the systemic circulation, and reach distant organs. Once inside host cells, they act as substrates, inhibitors, or cofactors for epigenetic enzymes, thereby directly influencing the host’s transcriptional landscape [39].

Emerging evidence after BS suggests that postoperative microbial remodeling occurs alongside broader metabolic and molecular adaptations involving microbial metabolites and host regulatory pathways. Human post-bariatric studies have reported postoperative changes in microbial composition and function, short-chain fatty acids, bile acid profiles, indole-related metabolites, DNA methylation, microRNAs, and metabolic gene expression. Importantly, integrated multi-omics studies have begun to examine these changes across interconnected biological levels, providing a broader view of the molecular response to BS. They suggested that BS is associated with parallel changes in microbiota, microbial metabolites, host DNA methylation, miRNAs, and metabolic gene expression. Nevertheless, the available evidence remains heterogeneous, and the associations observed between microbial, metabolic, and host molecular changes do not necessarily demonstrate direct mechanistic relationships. Furthermore, experimental and microbiota transfer studies provide additional support for the capacity of microbial communities and their metabolites to influence host metabolic and epigenetic pathways, but the relevance of these mechanisms to specific postoperative changes in humans requires further validation. Table 1 summarizes these findings across the major components of the proposed axis and highlights the distinction between evidence demonstrated after BS and complementary mechanistic evidence from experimental models (Table 1).

### 4.1. Short-Chain Fatty Acids (SCFAs) as Key Microbial Mediators

The main SCFAs produced in the human intestine are acetate, propionate, and butyrate. They are produced mainly by bacterial fermentation of dietary fiber but also come from the metabolism of organic and amino acids. Production has been observed to be strain-specific; for example, members of the Bacteroidetes phylum predominantly generate acetate and propionate, whereas Firmicutes species are the main producers of butyrate. Studies have observed significant changes in circulating SCFA levels 12 months following BS. Specifically, there was a marked increase in the levels of butyrate, propionate, isovalerate, and isobutyrate. Additionally, the greater rises in these SCFAs were associated with larger drops in body mass index [19].

Post-bariatric surgery, the enrichment of SCFA-producing bacteria contributes to the observed metabolic improvements. For instance, butyrate-mediated HDAC inhibition has been shown to upregulate the expression of genes involved in intestinal gluconeogenesis, which paradoxically helps improve systemic glucose homeostasis via a gut–brain neural circuit. Moreover, SCFAs can bind to and activate host cell G-protein-coupled receptors (GPRs), including GPR41 and GPR43. These receptors are also activated in adipose tissue, modulating adipokine secretion and reducing chronic inflammation, further impacting the host’s epigenetic and metabolic state [40].

Butyrate is especially important for gut health. It is an energy source for intestinal epithelial cells, stimulates the production of mucin, and encourages the proliferation of healthy colonocytes and has antiproliferative activity against cancerous colonocytes. In addition to barrier function, SCFAs regulate both innate and adaptive immunity. They promote the development and expansion of regulatory T cells, which are important for immune homeostasis; modulate B-cell metabolism to promote IgA production; and inhibit mast cell activation via butyrate and propionate. Collectively, these pleiotropic actions help maintain immune balance and tolerance to commensal microbes [39,41].

Beyond acting as energy sources for intestinal cells or interacting with G-protein-coupled receptors, butyrate is a strong natural inhibitor of both Class I and Class II histone deacetylases (HDACs). By inhibiting HDACs, butyrate promotes the hyperacetylation of histone lysine residues. This modification reduces the electrostatic attraction between histones and the negatively charged DNA backbone, resulting in a more relaxed and transcriptionally accessible chromatin structure. This mechanism allows for the activation of various genes involved in anti-inflammatory responses; cell growth, differentiation, and apoptosis; gut barrier maintenance; and metabolic regulation through the regulation of appetite, glucose, and lipid homeostasis [42,43,44].

Furthermore, butyrate exerts important effects on brain function by enhancing blood–brain barrier (BBB) integrity. It can cross the BBB and regulate neurotransmitter activity and neuroinflammatory responses while serving as a critical signaling molecule that facilitates bidirectional communication between the gut and the brain through the gut–brain axis [44].

### 4.2. Bile Acids: Signaling Through Nuclear Receptors

Secondary bile acids (BAs) have emerged as key regulators of the microbiome–epigenome interplay after BS, especially after malabsorptive BS like Roux-en-Y gastric bypass (RYGB). The changes that occur in the anatomy after the surgical reconfiguration allow the passage of concentrated bile acids to the distal intestine, where they interact with a remodeled gut microbiota. These changes alter the microbial capacity to deconjugate and dehydroxylate primary BAs into secondary BAs, such as deoxycholic acid (DCA) and lithocholic acid (LCA), which serve as preferential ligands for key metabolic receptors. Specifically, bile acids act as signaling molecules through receptors including the membrane G-protein-coupled receptor TGR5 and the nuclear receptor FXR. Several secondary BAs can activate TGR5, whereas FXR has a distinct ligand preference and is regulated by specific primary and secondary BAs. Therefore, the effects of bile acid signaling depend on the composition of the bile acid pool and the physiological context, with downstream pathways influencing glucose homeostasis, insulin sensitivity, energy metabolism, and other metabolic processes. FXR has been shown to recruit epigenetic modifiers, including the Class III histone deacetylase SIRT1 (a sirtuin). SIRT1 is a master metabolic regulator that deacetylates and activates key proteins such as PGC-1α (involved in mitochondrial biogenesis) while deacetylating and inhibiting lipogenic factors like SREBP-1c. This synergy between microbial-modified BAs and host sirtuins is a critical mechanism through which BS resets host lipid metabolism, suppresses fat accumulation, and resolves hepatic steatosis [20,21,22,23,24,30]. Concurrently, TGR5 activation in muscle and intestinal L-cells stimulates the secretion of metabolic hormones such as glucagon-like peptide-1 (GLP-1), which enhances resting energy expenditure and insulin sensitivity. Activation of these receptors leads to complex intracellular signaling pathways that eventually reach the nucleus of the cell, leading to significant remodeling of the epigenome without changing the genetic sequence. These signals trigger multiple changes in the epigenome through alterations in DNA methylation in metabolic tissues, including the liver, skeletal muscle, and adipose tissues. In patients with chronic obesity, genes responsible for healthy metabolism become silenced by obstructive methyl groups, while pro-inflammatory genes become highly expressed. Postoperative alterations in bile acid concentrations and signaling may contribute to changes in metabolic tissues through FXR-, TGR5-, and sirtuin-related pathways. Experimental evidence also suggests potential interactions between bile acid signaling and epigenetic regulators. However, the extent to which these pathways directly mediate epigenomic changes in human metabolic tissues after BS remains uncertain. In particular, evidence that postoperative bile acid changes directly alter DNA methylation at specific anti-obesity or insulin-signaling genes in humans is limited. Therefore, these pathways should be regarded as biologically plausible mechanisms rather than established causal pathways [20,21,22,23,24,30].

### 4.3. One-Carbon Metabolism and the Supply of Methyl Donors

The structural reconfiguration of the gastrointestinal tract following BS alters internal pH levels, shifts oxygen gradients, and modifies nutrient transit times. These dramatic changes lead to a rapid transformation of the gut microbiota, causing a massive expansion of specific beneficial bacterial phyla, most notably Bifidobacterium, Lactobacillus, and Bacillus subtilis [25,26,27,28,29,30,31,32].

This newly optimized microbiome experiences an immediate upregulation of functional genes dedicated to the de novo synthesis of critical micronutrients, providing a vital source of methyl donors such as folate, B12, and B6, which are essential for the host’s one-carbon metabolism. This metabolic network is responsible for the synthesis of S-adenosylmethionine (SAM), which serves as the universal methyl donor for DNA methyltransferases (DNMTs) and histone methyltransferases (HMTs). The sufficient amount of folate provided by microbiota can be used by the host to activate the methylation and suppression of pro-inflammatory cytokine genes such as TNF-α and IL-6 [25,26,27,28,29,30,31,32].

**Table 1:** Evidence supporting the gut microbiota, microbial metabolites, and host epigenetic regulation following bariatric surgery.

Microbial Component/Metabolite	Proposed Pathway	Evidence After Bariatric Surgery	Supporting Evidence from Other Models
Gut microbiota remodeling	DNA methylation and host gene expression	RYGB and SG are associated with persistent changes in microbial composition and functional potential, and microbial alterations are correlated with adipose tissue gene expression, inflammation, insulin sensitivity, and metabolic outcomes [12,45,46,47,48,49,50,51,52].	Preclinical studies support the biological relevance of microbiota remodeling after bariatric surgery, showing that surgically altered microbial communities can influence host energy metabolism and adiposity, with post-SG changes additionally linked to altered microbial bile acid metabolism [53,54].
SCFAs butyrate/propionate	Histone acetylation/HDAC activity	Postoperative studies demonstrate changes in circulating and/or fecal SCFAs, including butyrate, propionate, and acetate, that could be related to weight loss and improved insulin sensitivity [12,40,51,52,55,56]. However, direct links between these postoperative changes and histone acetylation have not been established.	Experimental studies demonstrate that SCFAs, particularly butyrate, can inhibit HDAC activity and modulate histone acetylation and gene expression [57,58].
Microbiota-derived bile acid metabolites	FXR/TGR5 signaling and downstream transcription	Bariatric surgery alters bile acid metabolism and signaling through FXR and TGR5, which may contribute to weight loss, improvements in glucose metabolism, and diabetes remission [24,48].	Preclinical studies demonstrate that altered microbiota and secondary bile acids contribute to the metabolic effects of SG/VSG through FXR and TGR5 signaling [54,59].
Microbial indole metabolites	AhR-associated transcriptional regulation	RYGB is associated with postoperative reductions in circulating tryptophan and downstream kynurenine pathway metabolites, with these changes correlating with BMI and HbA1c and accompanying improved glycemic control [40,48,50,60].	Experimental studies support biological effects of microbiota-derived indoles through AhR signaling [61,62].
Postoperative host DNA methylation remodeling	PPARGC1A, PDK4, PIK3R1, ITGB3, and other metabolic genes	RYGB and SG are associated with postoperative DNA methylation remodeling involving metabolically relevant pathways and genes including PPARGC1A, PDK4, PIK3R1, and ITGB3, with normalization toward levels observed in lean controls [63,64,65,66,67,68,69,70].	Experimental evidence supports epigenetic regulation of metabolic and inflammatory genes in obesity and weight loss models [71].
MicroRNAs	Post-transcriptional gene regulation	Bariatric surgery is associated with changes in circulating miRNAs related to improvement in BMI, glucose, lipid metabolism, inflammation, and liver enzymes toward levels observed in non-obese controls [16,72].	Experimental literature supports functional roles of specific miRNAs in metabolic and inflammatory pathways [73,74].
Integrated microbiota–metabolite–epigenome axis	DNA methylation, histone acetylation, miRNA regulation, and transcriptional signaling	Bariatric surgery is associated with parallel changes in microbiota, microbial metabolites, host DNA methylation, miRNAs, and metabolic gene expression [12,19,40,46,47,48,49,50,51,52,55,56,64,65,70].	Microbiota transfer and animal studies demonstrate that postoperative microbial communities can influence metabolic phenotype, while experimental models support effects of microbial metabolites on host signaling and epigenetic regulation [24,53,54,59,71].

Abbreviations: AhR, aryl hydrocarbon receptor; BMI, body mass index; FXR, farnesoid X receptor; HbA1c, glycated hemoglobin; HDAC, histone deacetylase; miRNA, microRNA; PDK4, pyruvate dehydrogenase kinase 4; PIK3R1, phosphoinositide-3-kinase regulatory subunit 1; PPARGC1A, peroxisome proliferator-activated receptor gamma coactivator 1-alpha; RYGB, Roux-en-Y gastric bypass; SCFAs, short-chain fatty acids; SG, sleeve gastrectomy; TGR5, Takeda G-protein-coupled receptor 5; VSG, vertical sleeve gastrectomy.

## 5. Epigenetic Remodeling of Host Metabolic Pathways

The positive metabolic effects of BS may involve tissue-specific epigenetic remodeling, alongside substantial anatomical, hormonal, metabolic, behavioral, and nutritional changes. Epigenomic changes in the host are mainly attributed to three interlinked processes: the process of DNA methylation that opens or silences gene promoters; the process of histone modification (which includes acetylation and methylation) that dynamically switches chromatin accessibility; and the expression of non-coding RNAs (microRNAs and long non-coding RNAs) that fine-tune metabolic transcripts. Ultimately, this remodeling process contributes to the regulation of glucose metabolism, lipid synthesis, and inflammatory responses across key metabolic tissues, including the liver, adipose tissue, and skeletal muscle [32,63,75].

### 5.1. Restoring Insulin Sensitivity

One of the notable impacts of BS is the relatively fast recovery of glucose control and possibility of remission of type 2 diabetes, which often happens within weeks or months after the surgery. The early improvement in glycemic control after BS is likely multifactorial and may involve changes in insulin signaling, gut hormones, bile acid metabolism, nutrient status, caloric intake, and potentially epigenetic regulation. Evidence suggests that changes in DNA methylation at genes involved in insulin signaling and glucose metabolism may occur following BS, including insulin receptor substrate 1 (IRS1) and glucose transporter 4 (GLUT4) in adipose tissue and skeletal muscle. Mechanistically, reduced methylation at regulatory regions may increase chromatin accessibility and facilitate the transcription of these genes. Increased IRS1 expression could enhance downstream insulin signaling pathways, including activation of phosphoinositide 3-kinase (PI3K) and protein kinase B (AKT), which are involved in insulin-mediated glucose uptake. Similarly, increased GLUT4 expression or translocation to the cell membrane could facilitate glucose uptake in skeletal muscle and adipose tissue. These changes may therefore contribute to improved insulin sensitivity after bariatric surgery. However, these mechanisms remain biologically plausible, and the available human evidence does not establish that surgery-induced methylation changes directly cause these metabolic effects [63]. Moreover, improvements have been reported in the epigenetic modification of the genes responsible for hepatic glucose production, including PEPCK, as well as in reducing excessive gluconeogenesis. These findings provide biological plausibility for a potential microbiome–epigenome–metabolism interaction; however, the available human evidence does not establish that these microbiome changes directly cause the observed epigenetic remodeling or improvement in insulin sensitivity.

In the case of skeletal muscle, it is worth mentioning that the decreased level of DNA methylation in the gene PPARGC1A encoding PGC-1α, the master regulator of mitochondrial biogenesis and oxidative metabolism, is linked to weight loss post-RYGB. Thus, enhanced expression of PGC-1α positively affects mitochondrial functionality [32,63,75].

Apart from the modifications that occur in DNA and histones, BS facilitates the reprogramming of body metabolism through the normalization of host microRNAs (miRNAs), which function as vital post-transcriptional regulators controlling the activity of gene expression within the microbiota–epigenome axis. The dramatic remodeling in the microbiome results in the establishment of a highly responsive, bidirectional communication loop. In this pathway, host-derived fecal miRNAs can directly enter bacterial cells to shape microbial composition, while the newly restructured gut microbiota reciprocally regulates host tissue miRNA expression. They target pathways regulating adipogenesis, insulin resistance, and liver lipid metabolism. In addition, the increase in Bacteroides and Parabacteroides correlates with precise shifts in hepatic miR-5112. This molecular shift turns on key genes such as Akt1, Socs3, and potassium calcium-activated channel subfamily N member 1 (Kcnn1) that help peripheral tissues to return to an insulin-sensitive state [76,77].

### 5.2. Adipose Tissue Remodeling and the Resolution of Inflammation

Obesity is associated with low-grade systemic inflammation, which is primarily due to the abnormal functioning of white adipose tissue (WAT). Obese individuals show a “pro-inflammatory epigenome”, wherein the promoters of inflammatory cytokines such as TNF and IL6 are hypomethylated, causing the upregulation of their expression. This condition is responsible for causing insulin resistance. BS results in extensive reprogramming of WAT epigenomes into anti-inflammatory and metabolically healthy states. Post-surgical weight loss promotes the de novo methylation of pro-inflammatory cytokine promoters, thereby switching off their expression and decreasing systemic inflammatory markers. WAT also secretes adiponectin, which acts as a powerful insulin sensitizer. Additionally, BS greatly changes the cell’s energetic state and thus activates metabolic regulators such as AMPK. This results in the reprogramming of chromatin-modifying enzymes to actively place open histone tags such as H3K27ac on mitochondrial and fatty acid oxidizing genes. Such targeted chromatin modification relaxes the structure of chromatin, exposing the OXPHOS genes. As a result, new fat cells are genetically programmed with a high oxidative capacity [78,79].

### 5.3. Hepatic Epigenetic Reprogramming

Bariatric surgeries have shown high effectiveness in resolving liver fat accumulation (hepatic steatosis), inflammation, and even liver fibrosis, which are closely related to hepatic epigenetic reprogramming. In particular, genes involved in de novo lipogenesis, such as FASN (fatty acid synthase) and SREBP-1c, become hypermethylated at their promoters, leading to reduced transcription and fat synthesis. On the contrary, genes that take part in fatty acid oxidation, like CPT1A, become less methylated in their promoters, leading to increased expression and higher rates of lipid degradation. Hepatic epigenetic modifications are greatly influenced by a different pool of metabolites generated by the microbiome post-surgery. As mentioned above, increased production of bile acids in the post-surgery gut enhances FXR activity and recruits SIRT1, which deacetylates and regulates transcription factors. Moreover, the decreased gut-derived inflammatory signals like LPS contribute to the resolution of liver inflammation and its associated epigenetic dysregulations. Additionally, the balanced microbiota following BS increases Natural Killer T (NKT) cells in the liver, preventing steatosis and inflammation in the organ. Interactions between the post-bariatric microbiome and metabolites may contribute to the hepatic regulatory pathways observed after BS. However, these interactions should not be considered the sole or established primary drivers of hepatic improvement, as BS simultaneously induces substantial changes in energy balance, nutrient availability, bile acid signaling, adiposity, and systemic inflammation [78,80,81,82,83].

Furthermore, the remarkable enrichment of specific groups of Bacteroidetes phyla following BS, especially Bacteroides uniformis, results in the regulation of miR-122 and the peroxisome proliferator-activated receptor alpha (PPARα), which consequently curbs excessive hepatic gluconeogenesis and downregulates tissue-level inflammation in the liver [76,77,84,85].

### 5.4. Cardiovascular Risk and Atherogenic Silencing

The process of recovery of the intestinal barrier after surgery leads to the deactivation of endothelial cells (first stage of blood vessel damage). The epigenetic reset of the vessel walls takes place due to the blocking of LPS and pro-inflammatory cytokines systemically. Histone modifications (for instance, H3K9ac) are removed from the promoters of vascular adhesion genes. This prevents accumulation of white blood cells in the blood vessels, thus inhibiting further development of atherosclerosis and decreasing the risk of cardiovascular disease. Additionally, a great reduction in fat mass, low-density lipoprotein, and total cholesterol levels was observed [12,86].

However, the rise in the level of trimethylamine N-oxide (TMAO) produced by gut microbiota after BS is rather surprising since the elevated level of TMAO is associated with an increased risk of developing CVD, while BS is known to reduce the risk of CVD. This surprising rise in a harmful bacterial metabolite shows that other protective mechanisms such as increased butyrate production and a healed gut barrier might be strong enough to override the TMAO effect [87,88].

## 6. Integration of Mechanistic Crosstalk: Intestinal Integrity and Tissue-Level Repair

The molecular mechanisms within the gut microbiota–epigenome axis do not work in isolation; instead, they form a highly connected loop that controls systemic metabolism. The basis of this crosstalk involves restoration of the integrity of the intestinal barrier so as to block metabolic endotoxemia (the leakage of bacterial toxins into the blood). Before BS, obesity-associated dysbiosis causes damage to the mucosal coating and downregulates structural cellular junctions in the intestine. In turn, this facilitates entry of lipopolysaccharide (LPS), an outer membrane component of Gram-negative bacteria and a major toxic component, allowing it to pass via paracellular pathways into the portal circulation. On entering the bloodstream, systemic LPS binds to Toll-Like Receptor 4 (TLR4) expressed on host immune cells, adipocytes, and hepatocytes and causes activation of NF-κB, a pro-inflammatory signaling cascade. This causes inflammatory signaling to alter the host epigenetic balance by upregulating pro-inflammatory cytokine genes and altering DNA methylation in metabolic pathways. BS interrupts this vicious cycle via an integrated biochemical process. This is because the increase in certain species of post-surgical bacteria such as Bacteroidetes and Lactobacillus causes an increase in SCFAs and indoles. These metabolites initiate epigenetic reprogramming locally within the intestinal epithelium, thus causing HDAC inhibition and recruiting histone acetyltransferases to the promoter region of important tight junction proteins such as Claudin-1, Occludin, and Zonula Occludens-1 (ZO-1) [9,38,89,90,91,92]. An illustration of the role of BS in driving mechanistic crosstalk within the gut microbiota–epigenome axis is presented in Figure 1.

## 7. Inter-Individual Weight Outcomes and Weight Regain

Changes in gut microbiota composition induced by BS have been proposed as potential determinants of weight loss and long-term metabolic improvements. Olsson and colleagues conducted a randomized controlled trial and found that both RYGB and SG result in microbiome shifts toward a functionally aligned profile; however, RYGB has larger compositional shifts and a more homogeneous gut microbiota [12]. Clinical remission is associated with increased bacterial gene richness, increased capability of methanogenesis, and expansion of several Akkermansia species. Importantly, clinical responders show a decrease in fecal SCFA levels (indicating increased host absorption), together with an increase in the branched-chain-to-short-chain fatty acid ratio (BCFA/SCFA), indicating faster SCFA absorption into the host tissue.

On the other hand, persistent differences in gut microbial composition or function after surgery may be associated with variability in metabolic responses, although the mechanisms underlying these differences remain incompletely understood. Some studies have reported associations between postoperative microbiota profiles, microbial metabolites, and clinical outcomes; however, these findings do not establish that microbiota changes directly determine weight loss or weight regain. Similarly, the concept of metabolic memory proposes that epigenetic alterations acquired during obesity may persist after subsequent metabolic improvement. Although such persistent epigenetic changes have been described in obesity and after weight loss, their specific contribution to postoperative weight regain has not been established in humans. Therefore, the proposed role of persistent “epigenetic scars” should currently be considered a mechanistic hypothesis requiring further investigation rather than an established explanation for variability in bariatric surgery outcomes [12,34,35].

## 8. Targeting the Microbiota–Epigenome Axis: Probiotic Evidence and Future Biotic Perspectives

### 8.1. Modulating the Axis via Biotic Therapeutics

Given the potential involvement of the gut microbiota in postoperative metabolic adaptation, microbiota-targeted interventions, including probiotics, have been investigated as possible adjunctive therapies after BS. However, current clinical evidence does not establish probiotics as effective modulators of the postoperative microbiota–epigenome axis. Previous meta-analysis studies published by Daghmouri et al., and Swierz et al., demonstrated no significant effect on weight-related outcomes. In contrast, there was a significant improvement in fasting insulin in T2DM patients and a significant decrease in triglyceride and LDL-cholesterol levels [93,94]. Additionally, probiotic supplementation (particularly from the Bifidobacterium and Lactobacillus genera) after BS led to statistically significant increases in serum concentrations of vitamins B12 and D, indicating that these strains function as active in vivo micro-factories that overcome malabsorption after surgery and restore the cofactor pool in the host. However, it did not significantly affect weight loss, metabolic parameters, or inflammatory markers [95]. In another study conducted by Sherf-Dagan et al., patients who underwent laparoscopic sleeve gastrectomy were administered high-dose, multi-strain probiotics (25 billion live bacteria per capsule with 11 different strains) for six months after surgery. The results of a direct analysis of fecal microbiome composition showed that there was no statistical difference in the diversity of microbial populations or the phyla present in the two groups at 3, 6, and 12 months after surgery. The only differences were temporary increases in the presence of Proteobacteria, Actinobacteria, and Collinsella at 6 months, which decreased again at 12 months. These results show that the anatomical changes of BS are the main driver of microbiome restructuring, leaving a relatively narrow therapeutic window for supplemental probiotics to induce permanent compositional shifts [96]. A summary of comparative human clinical evidence of microbiome-targeted interventions in bariatric surgery patients is presented in Table 2.

**Table 2:** Summary of comparative human clinical evidence of microbiome-targeted interventions in bariatric surgery.

Intervention	Definition	Outcome
Probiotics [93,94,95]	Living micro-organisms confer a health benefit when administered in adequate amounts.	No significant effect on weight-related outcomes. Significant improvement in vitamins D and B12, as well as fasting insulin in T2DM patients. Significant decrease in triglyceride and LDL-cholesterol levels.
Prebiotics [97]	Substrates selectively stimulate the growth of good bacteria already in the gut, promoting production of beneficial compounds.	Prebiotic supplementation group showed higher weight loss than both the placebo and synbiotic groups. Neither supplements produced statistically significant reductions in inflammatory markers.
Synbiotics [97]	A mixture comprising live microorganisms and substrate(s) selectively utilized by host microorganisms that confers a health benefit on the host.	
FMT [98]	The administration of human fecal matter containing a complex community of microorganisms from a healthy donor into the gastrointestinal tract of a recipient to restore or modify the gut microbiota.	FMT did not reduce the body weight of patients undergoing bariatric surgery. FMT may improve metabolic parameters, including insulin sensitivity, glucose metabolism, and inflammatory markers.

Abbreviations: FMT, fecal microbiota transplant; T2DM, type 2 diabetes mellitus; LDL, low-density lipoprotein.

Beyond the bariatric cohorts, a recent meta-analysis conducted by Guo and colleagues showed that probiotic supplementation can have beneficial effects on body weight parameters. The decrease in weight, visceral fat content, and waist circumference in the experimental group taking probiotics was significantly higher than that in the placebo group. However, there was no significant difference in BMI and LDL-C levels between the experimental group and the control group [99].

Furthermore, evidence suggests that the potential anti-obesity effects of probiotics are highly strain-specific. Certain strains of Lactobacillus (including *L. casei*, *L. rhamnosus*, *L. gasseri*, and *L. plantarum*) and Bifidobacterium (including *B. longum*, *B. animalis*, and *B. breve*) have demonstrated associations with reduced body weight or adiposity in both preclinical and clinical studies [100].

### 8.2. Current Limitations and Therapeutic Barriers

The characterization of probiotics as definitive clinical modulators of the post-bariatric microbiota–epigenome axis remains limited by several major methodological challenges in the current literature. First, there is a notable lack of direct tissue-based epigenetic analyses. Existing clinical trials have primarily focused on conventional anthropometric and metabolic outcomes, without incorporating paired, high-resolution epigenomic sequencing approaches to determine whether probiotic interventions induce functional alterations in host gene regulation. Second, substantial heterogeneity exists among available studies, including variations in probiotic strains, formulations, dosages, intervention durations, and measured outcomes. Furthermore, the predominance of multi-strain probiotic preparations limits the ability to define strain-specific effects, resulting in inconsistent findings and emphasizing the need for further research to identify the probiotic strains with the greatest potential for modulating body weight and metabolic outcomes. Importantly, associations between probiotic supplementation, changes in microbial composition, and clinical outcomes should not be interpreted as evidence that microbiota modulation directly causes the observed metabolic or nutritional effects. Demonstrating causality will require trials incorporating longitudinal microbiome profiling, metabolomics, and appropriate host molecular measurements alongside clinical outcomes.

Future studies should include larger cohorts with extended follow-up periods and integrated multi-omics approaches. Given that epigenetic modifications represent long-term adaptive mechanisms involved in host metabolic regulation, short-term studies may be insufficient to determine whether microbiota-targeted interventions result in sustained or durable alterations in metabolic programming [101,102,103].

## 9. Emerging Multi-Omics Approaches

In order to understand and decode the post-bariatric microbiota–epigenome axis comprehensively, research is moving beyond just studying single-layer profiling and toward a multi-omics approach. The combination of detailed sequencing of microbial species, transcriptional analysis of activity, and systemic metabolite tracking with host tissue epigenomics provides scientists with a way of identifying the pathways that link specific probiotic strains to chromatin alterations. Moreover, an important insight emerging from the current literature is that the most informative biomarker after BS may not be a microbial taxon itself, but rather a functional signal linking microbial activity to host physiology. Taxonomic changes are highly variable across studies and may reflect differences in diet, medications, surgical procedure, geography, and baseline metabolic status. Consequently, identifying a bacterium that increases or decreases after surgery does not, by itself, establish its biological importance. A more meaningful sequence is to determine whether a microbial functional change produces an altered metabolite profile, whether these metabolites are associated with changes in host metabolic or epigenetic pathways, and whether these molecular changes precede and predict subsequent clinical improvement. Accordingly, we propose that future bariatric studies should adopt longitudinal multi-omics designs with repeated microbiome, metabolomic, metabolic, and epigenomic measurements, together with detailed dietary and medication data and clinically meaningful long-term outcomes. Details of these emerging multi-omics approaches, their biological targets, and their contributions to epigenetic discovery are shown in Table 3 [104,105,106,107].

## 10. Bariatric Surgery Versus Emerging Pharmacological Therapies

Recent pharmacological advances have narrowed the efficacy gap between medical and surgical treatment of obesity. Tirzepatide (Zepbound), a dual GIP/GLP-1 receptor agonist, produced approximately 15–21% mean weight loss after 72 weeks at doses of 5–15 mg in adults with obesity or overweight without diabetes, with dose escalation during the first 20 weeks. Adverse events caused treatment discontinuation in 4.3%, 7.1%, 6.2%, and 2.6% of participants receiving 5 mg, 10 mg, and 15 mg tirzepatide doses [108]. Moreover, Retatrutide, a triple GIP/GLP-1/glucagon receptor agonist, resulted in 24.2% mean weight loss after 48 weeks at 12 mg in a phase 2 trial, as compared with 2.1% in the placebo group. These results should not be interpreted as direct comparisons with BS because the trials differed in treatment duration, dose escalation, adherence and completion, eligibility criteria, diabetes status, and lifestyle interventions. Importantly, the magnitude of pharmacological weight loss must also be considered alongside treatment persistence and long-term maintenance. Although newer incretin-based therapies offer promising alternatives to BS, whether their benefits can match the long-term and broader metabolic effects of surgery remains uncertain [109].

## 11. Current Challenges and Knowledge Gaps

Translating the gut microbiota–epigenome axis from bench to bedside following BS is associated with practical difficulties. One of the key problems in the current available literature is the insufficient number of samples in most post-surgical studies. Additionally, the observed heterogeneity was high in many studies for most of the outcomes. According to Debédat et al., most of the variations in the studies’ findings can be attributed to various technical and external differences. From a technical perspective, researchers employ different techniques for DNA extraction and sequencing that include not only simple techniques like qPCR or lower-resolution marker sequencing (16S rRNA) but also highly accurate whole-genome shotgun sequencing. Apart from that, there are significant time differences between sample collection. Although the 16S sequencing technique is widely used, it does not provide information about specific bacterial strains, and it lacks the resolution to map functional microbial genes responsible for SCFA or B vitamin synthesis [110,111,112,113].

Critical methodological gaps also limit our mechanistic understanding of this axis. Target tissue biopsies (such as visceral adipose tissue or the liver) are not typically obtained from patients in parallel in human studies. Consequently, researchers have to utilize stool samples and peripheral blood mononuclear cells, which do not accurately reflect chromatin states in metabolic organs. Moreover, most of the available data in humans are observational in nature. It is necessary to track isotope-labeled metabolites in humans or use humanized models in order to determine whether a particular host DNA methylation change is a result of microbial metabolites or caloric restriction. Furthermore, the available human literature on the gut microbiota, epigenetic regulation, and metabolic outcomes following BS remains observational or mechanistic rather than directly causal. Experimental and mechanistic studies provide important biological plausibility, but the independent causal contribution of the microbiome and its metabolites to postoperative epigenetic and metabolic remodeling remains incompletely defined in humans. Finally, studies evaluating probiotic benefits after BS usually focus on clinical endpoints (like weight loss or serum lipids) rather than performing serial stool metagenomics or metabolomics, leaving the actual composition and metabolite production of the supplemented strains unverified [110,111].

## 12. Future Perspectives

Future research should move beyond conventional anthropometric measures toward a precision bariatric medicine approach that integrates gut microbiota, microbial metabolites, epigenetic biomarkers, and clinical phenotypes to better characterize inter-individual variability in postoperative outcomes. Whereas existing bariatric metrics primarily focus on the percentage of excess weight loss or BMI reduction, future studies are suggested to focus on measuring the efficacy of surgery by employing epigenetic aging algorithms such as Horvath’s clock and GrimAge. Monitoring post-bariatric alterations in gut microbiota-derived metabolites, such as increased butyrate production and methyl donor availability, may provide mechanistic insights into host epigenetic remodeling and markers of DNA methylation aging following bariatric surgery. These biomarkers could offer a more comprehensive assessment of metabolic health and cardiovascular risk than body weight alone, although their clinical utility requires further validation. Additionally, future studies should validate the survival and functional activity of probiotic strains within the surgically altered gastrointestinal environment, characterized by changes in pH and intestinal transit time, while tailoring conventional and next-generation probiotic interventions according to each patient’s preoperative microbial and epigenetic profile. Moreover, to overcome the challenges associated with live bacterial colonization after surgery, postbiotics, comprising purified microbial components or bioactive metabolites such as SCFAs, represent a promising therapeutic alternative. Furthermore, the development of epigenetic biomarkers to predict postoperative weight regain and guide clinical decision-making, together with the investigation of fecal microbiota transplantation from patients with favorable postoperative responses to those with suboptimal outcomes, may further advance microbiota-based therapeutic strategies and accelerate the transition toward personalized bariatric care [12,34,35,93,94,98].

## 13. Conclusions

Growing evidence indicates that BS is accompanied by coordinated changes in gut microbial composition and function, microbial metabolites, and host metabolic and regulatory pathways. These changes provide a potential framework for understanding how postoperative microbial remodeling may interact with host processes involved in metabolic adaptation. Microbial metabolites, including SCFAs, bile acid derivatives, and indole-related compounds, can influence host signaling and gene regulation through pathways involving histone deacetylases, DNA methylation, nuclear and G-protein-coupled receptors, and inflammatory signaling. However, the extent to which these mechanisms contribute directly to postoperative epigenetic remodeling in humans remains incompletely established.

Current clinical evidence suggests that postoperative probiotic supplementation may improve selected nutritional or metabolic outcomes, including vitamin B12 and D concentrations and some lipid or glycemic measures, but does not consistently provide additional weight loss benefit. The available evidence is also limited by heterogeneity in probiotic strains, doses, treatment duration, patient populations, and outcome assessment, as well as limited direct assessment of microbiome function and host epigenetic changes.

Future research should therefore prioritize adequately powered longitudinal randomized trials integrating microbiome profiling, metabolomics, and tissue- or blood-based epigenetic measurements with clinically meaningful outcomes. Such studies are needed to determine whether microbiota-derived metabolites and epigenetic alterations are mediators of postoperative metabolic improvement or simply correlate with broader changes induced by surgery and weight loss. Postbiotics, personalized probiotic strategies, fecal microbiota transplantation, and epigenetic biomarkers represent promising areas for investigation, but their clinical effectiveness remains to be established. Ultimately, a better understanding of the microbiota–epigenome relationship may contribute to precision bariatric care, provided that mechanistic hypotheses are supported by rigorous longitudinal and interventional human evidence.

## Figures and Tables

**Figure 1:**
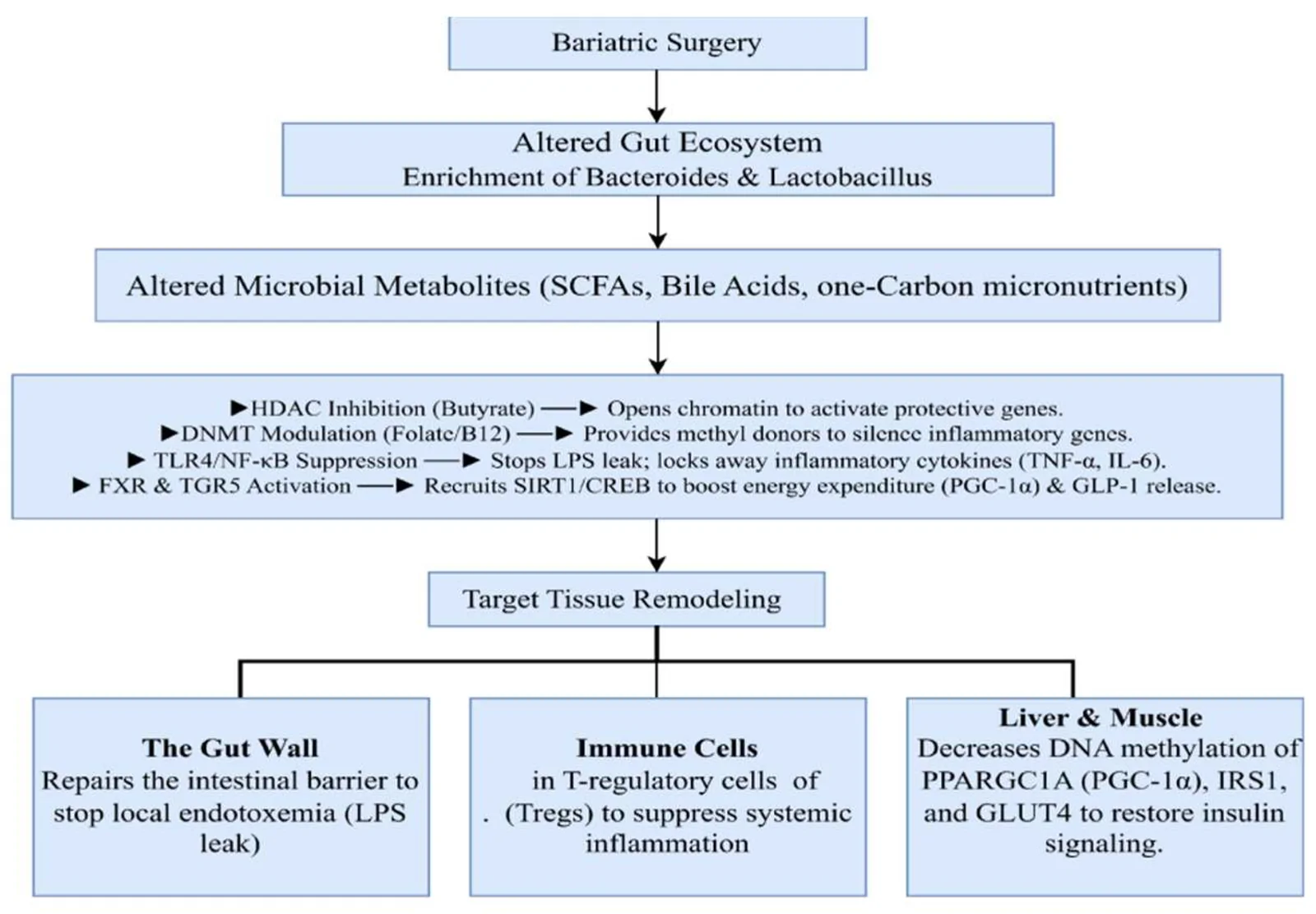
Mechanistic flow of the gut microbiota–epigenome axis and tissue-level remodeling post-bariatric surgery. Abbreviations: SCFAs, short-chain fatty acids; HDAC, histone deacetylase; DNMT, DNA methyltransferase; TLR4, Toll-Like Receptor 4; NF-κB, nuclear factor kappa-light-chain-enhancer of activated B cells; LPS, lipopolysaccharide; TNF-α, tumor necrosis factor-alpha; IL-6, Interleukin-6; FXR, farnesoid X receptor; TGR5, Takeda G-protein 5; SIRT1, silent information regulator (Sirtuin) 1; CREB, cAMP response element-binding protein; PGC-1α, peroxisome proliferator-activated receptor gamma coactivator 1-alpha; GLP-1, glucagon-like peptide-1; PPARGC1A, peroxisome proliferator-activated receptor gamma coactivator 1-alpha gene; IRS1, insulin receptor substrate 1; GLUT4, glucose transporter type 4.

**Table 3:** Emerging multi-omics approaches in gut microbiota–epigenome research [75,76,77,78].

Omics Approach	Description	Role in Microbiota–Epigenome Axis
Metagenomics	Sequencing and analysis of all microbial DNA within a sample to characterize microbial composition and functional potential.	Identifies microbial composition and diversity. Detects genes involved in metabolite production. Predicts microbial functional potential. Identifies taxa associated with epigenetic changes.
Metabolomics	Comprehensive analysis of small-molecule metabolites in biological samples.	Quantifies microbial metabolites. Identifies epigenetically active metabolites. Links metabolites to host metabolic pathways.
Metatranscriptomics	Analysis of the collective microbial RNA transcripts in a biological sample.	Identifies actively expressed microbial genes. Reveals functional microbial activity. Links gene expression to metabolite production.
Epigenomics	Genome-wide analysis of epigenetic modifications affecting gene expression.	Profiles DNA methylation and histone modifications. Identifies microbiota-associated epigenetic changes. Reveals host gene regulatory alterations.
Multi-Omics Integration	Integration of multiple omics datasets to provide a comprehensive view of biological systems.	Integrates microbial, metabolite, and epigenetic data. Identifies host–microbiota interaction networks. Reveals mechanistic pathways.
AI and Systems Biology	Computational tools for pattern recognition and system modeling.	Integrates complex multi-omics datasets. Predicts microbiota–epigenome interactions. Identifies biomarkers and therapeutic targets.

Abbreviations: AI, Artificial Intelligence.

## Data Availability

No new data were created or analyzed in this study.

## Abbreviations

The following abbreviations are used in this manuscript:

AhR	Aryl hydrocarbon receptor
BAs	Bile acids
BMI	Body mass index
BS	Bariatric surgery
DNMTs	DNA methyltransferases
FMT	Fecal microbiota transplantation
FXR	Farnesoid X receptor
GI	Gastrointestinal
GLP-1	Glucagon-like peptide-1
GPR	G-protein-coupled receptor
HATs	Histone acetyltransferases
HDACs	Histone deacetylases
HMTs	Histone methyltransferases
RYGB	Roux-en-Y gastric bypass
SAM	S-adenosylmethionine
SCFAs	Short-chain fatty acids
SG	Sleeve gastrectomy
TGR5	Takeda G-protein-coupled receptor 5
TLR4	Toll-Like Receptor 4
TMAO	Trimethylamine N-oxide

## References

[1] GBD 2021 Risk Factors Collaborators (2024). Global burden and strength of evidence for 88 risk factors in 204 countries and 811 subnational locations, 1990–2021: A systematic analysis for the Global Burden of Disease Study 2021. Lancet.

[2] GBD 2021 Adult BMI Collaborators (2025). Global, regional, and national prevalence of adult overweight and obesity, 1990–2021, with forecasts to 2050: A forecasting study for the Global Burden of Disease Study 2021. Lancet.

[3] Mahmoud Marey M., Alkilani F., Mahmoud Genidy A., Mahmood J., Adel M., M. Elhady M. (2025). Thyroid hormone receptor beta (THRβ) Agonists in Metabolic Dysfunction-Associated Steatohepatitis (MASH): Bridging the Gap Between Promise and Practice. J. Gastrointestin. Liver Dis..

[4] Dall T.M., Sapra T., Natale Z., Livingston T., Chen F. (2024). Assessing the economic impact of obesity and overweight on employers: Identifying opportunities to improve work force health and well-being. Nutr. Diabetes.

[5] Bariatric Surgery Procedures—American Society for Metabolic and Bariatric Surgery. https://asmbs.org/patients/bariatric-surgery-procedures/.

[6] Awad A.A., Abdrabou Abouelmagd A., Mohammed F., Elettreby A.M., Mahmoud Marey M., Aldemerdash M.A., Soliman S.M., Belal M.M., Abosheaishaa H. (2025). Evaluating the efficacy and safety of survodutide for obesity: A systematic review and meta-analysis of randomized controlled trials. Bayl. Univ. Med. Cent. Proc..

[7] Bariatric Surgery Significantly More Effective Than GLP-1 Drugs for Weight Loss and Disease Remission, Large Real-world Study Finds—American Society for Metabolic and Bariatric Surgery. https://asmbs.org/news_releases/bariatric-surgery-significantly-more-effective-than-glp-1-drugs-for-weight-loss-and-disease-remission-large-real-world-study-finds/.

[8] Risks of Diabetes Surgery no Higher than Commonly Performed Surgeries|ScienceDaily. https://www.sciencedaily.com/releases/2014/11/141106082036.htm.

[9] Kaplan U., Zohdy W., Gmora S., Hong D., Anvari M. (2022). What patient factors influence bariatric surgery outcomes? A multiple regression analysis of Ontario Bariatric Registry data. Can. J. Surg..

[10] Bruinsma F.F.E., Nienhuijs S.W., Liem R.S.L., Greve J.W.M., Marang-van de Mheen P.J., Dutch Audit for Treatment of Obesity Research Group (2025). Variation in outcomes after metabolic bariatric surgery: Multilevel analysis to assess the contribution of patient, surgeon, and hospital factors. Br. J. Surg..

[11] Robinson A.H., Adler S., Stevens H.B., Darcy A.M., Morton J.M., Safer D.L. (2014). What variables are associated with successful weight loss outcomes for bariatric surgery after 1 year?. Surg. Obes. Relat. Dis..

[12] Olsson L.M., Borgeraas H., Chakaroun R.M., Hofsø D., Hertel J.K., Dwibedi C., Mitteregger M., Holst J.J., Tremaroli V., Hjelmesæth J. (2026). Gut microbiota responses to bariatric surgery are associated with metabolic outcomes and type 2 diabetes remission. Nat. Metab..

[13] Aron-Wisnewsky J., Prifti E., Belda E., Ichou F., Kayser B.D., Dao M.C., Verger E.O., Hedjazi L., Bouillot J.-L., Chevallier J.-M. (2019). Major microbiota dysbiosis in severe obesity: Fate after bariatric surgery. Gut.

[14] Cotillard A., Kennedy S.P., Kong L.C., Prifti E., Pons N., Le Chatelier E., Almeida M., Quinquis B., Levenez F., Galleron N. (2013). Dietary intervention impact on gut microbial gene richness. Nature.

[15] Blüher M. (2014). Are metabolically healthy obese individuals really healthy?. Eur. J. Endocrinol..

[16] Izquierdo A.G., Crujeiras A.B. (2019). Obesity-Related Epigenetic Changes After Bariatric Surgery. Front. Endocrinol..

[17] Shreiner A.B., Kao J.Y., Young V.B. (2015). The gut microbiome in health and in disease. Curr. Opin. Gastroenterol..

[18] Kato K. (2022). What is epigenetics?. Inflammation and Oral Cancer.

[19] Martínez-Sánchez M.A., Balaguer-Román A., Fernández-Ruiz V.E., Almansa-Saura S., García-Zafra V., Ferrer-Gómez M., Frutos M.D., Queipo-Ortuño M.I., Ruiz-Alcaraz A.J., Núñez-Sánchez M.Á. (2023). Plasma short-chain fatty acid changes after bariatric surgery in patients with severe obesity. Surg. Obes. Relat. Dis..

[20] Lee C.J., Florea L., Sears C.L., Maruthur N., Potter J.J., Schweitzer M., Magnuson T., Clark J.M. (2019). Changes in Gut Microbiome after Bariatric Surgery Versus Medical Weight Loss in a Pilot Randomized Trial. Obes. Surg..

[21] Liou A.P., Paziuk M., Luevano J.-M., Machineni S., Turnbaugh P.J., Kaplan L.M. (2013). Conserved shifts in the gut microbiota due to gastric bypass reduce host weight and adiposity. Sci. Transl. Med..

[22] Kaska L., Sledzinski T., Chomiczewska A., Dettlaff-Pokora A., Swierczynski J. (2016). Improved glucose metabolism following bariatric surgery is associated with increased circulating bile acid concentrations and remodeling of the gut microbiome. World J. Gastroenterol..

[23] Homs E., Vives M., Torrell H., Castillo D.D., Sánchez A., Sabench F. (2026). Impact of bariatric surgery on the gut microbiota and metabolomic profile of the enterohepatic axis in an experimental model of obesity. Cir. Esp..

[24] Wahlström A., Aydin Ö., Olsson L.M., Sjöland W., Henricsson M., Lundqvist A., Marschall H.-U., Franken R., van de Laar A., Gerdes V. (2024). Alterations in bile acid kinetics after bariatric surgery in patients with obesity with or without type 2 diabetes. EBioMedicine.

[25] Bozack A.K., Khodasevich D., Nwanaji-Enwerem J.C., Gladish N., Shen H., Daredia S., Gamble M., Needham B.L., Rehkopf D.H., Cardenas A. (2025). One-carbon metabolism-related compounds are associated with epigenetic aging biomarkers: Results from the cross-sectional National Health and Nutrition Examination Survey 1999–2002. Am. J. Clin. Nutr..

[26] Liu J.J., Ward R.L. (2010). Folate and one-carbon metabolism and its impact on aberrant DNA methylation in cancer. Adv. Genet..

[27] Woting A., Blaut M. (2016). The intestinal microbiota in metabolic disease. Nutrients.

[28] Haran A., Bergel M., Kleiman D., Hefetz L., Israeli H., Weksler-Zangen S., Agranovich B., Abramovich I., Ben-Haroush Schyr R., Gottlieb E. (2023). Differential effects of bariatric surgery and caloric restriction on hepatic one-carbon and fatty acid metabolism. iScience.

[29] Friso S., Udali S., De Santis D., Choi S.-W. (2017). One-carbon metabolism and epigenetics. Mol. Asp. Med..

[30] Metere A., Graves C.E. (2020). Factors influencing epigenetic mechanisms: Is there A role for bariatric surgery?. High-Throughput.

[31] Hamamah S., Hajnal A., Covasa M. (2024). Influence of bariatric surgery on gut microbiota composition and its implication on brain and peripheral targets. Nutrients.

[32] Lin X., Han H., Wang N., Wang C., Qi M., Wang J., Liu G. (2024). The Gut Microbial Regulation of Epigenetic Modification from a Metabolic Perspective. Int. J. Mol. Sci..

[33] Zhang Q., Liu Y., Li Y., Bai G., Pang J., Wu M., Li J., Zhao X., Xia Y. (2025). Implications of gut microbiota-mediated epigenetic modifications in intestinal diseases. Gut Microbes.

[34] Faria S.L., Santos A., Magro D.O., Cazzo E., Assalin H.B., Guadagnini D., Vieira F.T., Dutra E.S., Saad M.J.A., Ito M.K. (2020). Gut Microbiota Modifications and Weight Regain in Morbidly Obese Women After Roux-en-Y Gastric Bypass. Obes. Surg..

[35] Palmisano S., Campisciano G., Silvestri M., Guerra M., Giuricin M., Casagranda B., Comar M., de Manzini N. (2020). Changes in Gut Microbiota Composition after Bariatric Surgery: A New Balance to Decode. J. Gastrointest. Surg..

[36] Xu W., Yu J., Yang Y., Li Z., Zhang Y., Zhang F., Wang Q., Xie Y., Zhao B., Wu C. (2023). Strain-level screening of human gut microbes identifies Blautia producta as a new anti-hyperlipidemic probiotic. Gut Microbes.

[37] Santos J.M., Mathew M.S., Shah N., Pajuelo-Vasquez R., Mistry A.M., Heindl S.E. (2021). Pre and Post-Operative Alterations of the Gastrointestinal Microbiome Following Bariatric Surgery. Cureus.

[38] Zambrano A.K., Paz-Cruz E., Ruiz-Pozo V.A., Cadena-Ullauri S., Tamayo-Trujillo R., Guevara-Ramírez P., Zambrano-Villacres R., Simancas-Racines D. (2024). Microbiota dynamics preceding bariatric surgery as obesity treatment: A comprehensive review. Front. Nutr..

[39] Chaudhari S.N., Luo J.N., Harris D.A., Aliakbarian H., Yao L., Paik D., Subramaniam R., Adhikari A.A., Vernon A.H., Kiliç A. (2021). A microbial metabolite remodels the gut-liver axis following bariatric surgery. Cell Host Microbe.

[40] Yu D., Shu X.-O., Howard E.F., Long J., English W.J., Flynn C.R. (2020). Fecal metagenomics and metabolomics reveal gut microbial changes after bariatric surgery. Surg. Obes. Relat. Dis..

[41] Stein R.A., Riber L. (2023). Epigenetic effects of short-chain fatty acids from the large intestine on host cells. microLife.

[42] Everard A., Belzer C., Geurts L., Ouwerkerk J.P., Druart C., Bindels L.B., Guiot Y., Derrien M., Muccioli G.G., Delzenne N.M. (2013). Cross-talk between Akkermansia muciniphila and intestinal epithelium controls diet-induced obesity. Proc. Natl. Acad. Sci. USA.

[43] Bridgeman S., Ellison G., Newsholme P., Mamotte C. (2021). The HDAC inhibitor butyrate impairs β cell function and activates the disallowed gene hexokinase I. Int. J. Mol. Sci..

[44] Chakraborty P., Laird A.S. (2025). Understanding activity of butyrate at a cellular level. Neural Regen. Res..

[45] Furet J.-P., Kong L.-C., Tap J., Poitou C., Basdevant A., Bouillot J.-L., Mariat D., Corthier G., Doré J., Henegar C. (2010). Differential Adaptation of Human Gut Microbiota to Bariatric Surgery-Induced Weight Loss: Links with Metabolic and Low-Grade Inflammation Markers. Diabetes.

[46] Kong L.-C., Tap J., Aron-Wisnewsky J., Pelloux V., Basdevant A., Bouillot J.-L., Zucker J.-D., Doré J., Clément K. (2013). Gut Microbiota after Gastric Bypass in Human Obesity: Increased Richness and Associations of Bacterial Genera with Adipose Tissue Genes. Am. J. Clin. Nutr..

[47] Palleja A., Kashani A., Allin K.H., Nielsen T., Zhang C., Li Y., Brach T., Liang S., Feng Q., Jørgensen N.B. (2016). Roux-En-Y Gastric Bypass Surgery of Morbidly Obese Patients Induces Swift and Persistent Changes of the Individual Gut Microbiota. Genome Med..

[48] Ilhan Z.E., DiBaise J.K., Dautel S.E., Isern N.G., Kim Y.-M., Hoyt D.W., Schepmoes A.A., Brewer H.M., Weitz K.K., Metz T.O. (2020). Temporospatial Shifts in the Human Gut Microbiome and Metabolome after Gastric Bypass Surgery. npj Biofilms Microbiomes.

[49] Dang J.T., Mocanu V., Park H., Laffin M., Hotte N., Karmali S., Birch D.W., Madsen K.L. (2022). Roux-En-Y Gastric Bypass and Sleeve Gastrectomy Induce Substantial and Persistent Changes in Microbial Communities and Metabolic Pathways. Gut Microbes.

[50] Penney N.C., Yeung D.K.T., Garcia-Perez I., Posma J.M., Kopytek A., Garratt B., Ashrafian H., Frost G., Marchesi J.R., Purkayastha S. (2022). Multi-Omic Phenotyping Reveals Host-Microbe Responses to Bariatric Surgery, Glycaemic Control and Obesity. Commun. Med..

[51] Martínez-Montoro J.I., Martín-Núñez G.M., González-Jiménez A., Garrido-Sánchez L., Moreno-Indias I., Tinahones F.J. (2024). Interactions between the Gut Microbiome and DNA Methylation Patterns in Blood and Visceral Adipose Tissue in Subjects with Different Metabolic Characteristics. J. Transl. Med..

[52] Juárez-Fernández M., Román-Sagüillo S., Porras D., García-Mediavilla M.V., Linares P., Ballesteros-Pomar M.D., Urioste-Fondo A., Álvarez-Cuenllas B., González-Gallego J., Sánchez-Campos S. (2021). Long-Term Effects of Bariatric Surgery on Gut Microbiota Composition and Faecal Metabolome Related to Obesity Remission. Nutrients.

[53] Tremaroli V., Karlsson F., Werling M., Ståhlman M., Kovatcheva-Datchary P., Olbers T., Fändriks L., le Roux C.W., Nielsen J., Bäckhed F. (2015). Roux-en-Y Gastric Bypass and Vertical Banded Gastroplasty Induce Long-Term Changes on the Human Gut Microbiome Contributing to Fat Mass Regulation. Cell Metab..

[54] Zhao S., Lin H., Li W., Xu X., Wu Q., Wang Z., Shi J., Chen Y., Ye L., Xi L. (2025). Post Sleeve Gastrectomy-Enriched Gut Commensal Clostridia Promotes Secondary Bile Acid Increase and Weight Loss. Gut Microbes.

[55] Meijer J.L., Roderka M.N., Chinburg E.L., Renier T.J., McClure A.C., Rothstein R.I., Barry E.L., Billmeier S., Gilbert-Diamond D. (2022). Alterations in Fecal Short-Chain Fatty Acids after Bariatric Surgery: Relationship with Dietary Intake and Weight Loss. Nutrients.

[56] Ahmad A.F., Caparrós-Martín J.A., Lee S., O’Gara F., Yeap B.B., Green D.J., Ballal M., Ward N.C., Dwivedi G. (2023). Gut Microbiome and Associated Metabolites Following Bariatric Surgery and Comparison to Healthy Controls. Microorganisms.

[57] Fellows R., Denizot J., Stellato C., Cuomo A., Jain P., Stoyanova E., Balázsi S., Hajnády Z., Liebert A., Kazakevych J. (2018). Microbiota Derived Short Chain Fatty Acids Promote Histone Crotonylation in the Colon through Histone Deacetylases. Nat. Commun..

[58] Donohoe D.R., Collins L.B., Wali A., Bigler R., Sun W., Bultman S.J. (2012). The Warburg Effect Dictates the Mechanism of Butyrate-Mediated Histone Acetylation and Cell Proliferation. Mol. Cell.

[59] Ryan K.K., Tremaroli V., Clemmensen C., Kovatcheva-Datchary P., Myronovych A., Karns R., Wilson-Pérez H.E., Sandoval D.A., Kohli R., Bäckhed F. (2014). FXR Is a Molecular Target for the Effects of Vertical Sleeve Gastrectomy. Nature.

[60] Yeung K.T.D., Penney N., Whiley L., Ashrafian H., Lewis M.R., Purkayastha S., Darzi A., Holmes E. (2022). The impact of bariatric surgery on serum tryptophan-kynurenine pathway metabolites. Sci. Rep..

[61] Hou Y., Li J., Ying S. (2023). Tryptophan metabolism and gut microbiota: A novel regulatory axis integrating the microbiome, immunity, and cancer. Metabolites.

[62] Jin U.-H., Lee S.-O., Sridharan G., Lee K., Davidson L.A., Jayaraman A., Chapkin R.S., Alaniz R., Safe S. (2014). Microbiome-Derived Tryptophan Metabolites and Their Aryl Hydrocarbon Receptor-Dependent Agonist and Antagonist Activities. Mol. Pharmacol..

[63] Pinhel M.A.S., Noronha N.Y., Nicoletti C.F., Pereira V.A., de Oliveira B.A., Cortes-Oliveira C., Salgado W., Barbosa F., Marchini J.S., Souza D.R. (2020). Changes in DNA Methylation and Gene Expression of Insulin and Obesity-Related Gene PIK3R1 after Roux-en-Y Gastric Bypass. Int. J. Mol. Sci..

[64] Barres R., Kirchner H., Rasmussen M., Yan J., Kantor F.R., Krook A., Näslund E., Zierath J.R. (2013). Weight Loss after Gastric Bypass Surgery in Human Obesity Remodels Promoter Methylation. Cell Rep..

[65] Bouchard-Mercier A., de Toro-Martín J., Nadeau M., Lescelleur O., Lebel S., Richard D., Biertho L., Tchernof A., Vohl M.-C. (2022). Molecular Remodeling of Adipose Tissue Is Associated with Metabolic Recovery after Weight Loss Surgery. J. Transl. Med..

[66] Nilsson E.K., Ernst B., Voisin S., Almén M.S., Benedict C., Mwinyi J., Fredriksson R., Schultes B., Schiöth H.B. (2015). Roux-En Y Gastric Bypass Surgery Induces Genome-Wide Promoter-Specific Changes in DNA Methylation in Whole Blood of Obese Patients. PLoS ONE.

[67] Nicoletti C.F., Pinhel M.A.S., Diaz-Lagares A., Casanueva F.F., Jácome A., Pinhanelli V.C., de Oliveira B.A.P., Crujeiras A.B., Nonino C.B. (2019). DNA Methylation Screening after Roux-En Y Gastric Bypass Reveals the Epigenetic Signature Stems from Genes Related to the Surgery per Se. BMC Med. Genom..

[68] Garcia L.A., Day S.E., Coletta R.L., Campos B., Benjamin T.R., De Filippis E., Madura J.A., Mandarino L.J., Roust L.R., Coletta D.K. (2021). Weight Loss after Roux-En-Y Gastric Bypass Surgery Reveals Skeletal Muscle DNA Methylation Changes. Clin. Epigenet..

[69] Coppedè F., Seghieri M., Stoccoro A., Santini E., Giannini L., Rossi C., Migliore L., Solini A. (2019). DNA Methylation of Genes Regulating Appetite and Prediction of Weight Loss after Bariatric Surgery in Obese Individuals. J. Endocrinol. Investig..

[70] Agodi A., Ojeda-Granados C., Maugeri A., Barchitta M., Coco O., Pezzino S., Magro G., Greca G.L., Latteri F.S., Castorina S. (2024). Changes in Gut Microbial Composition and DNA Methylation in Obese Patients with NAFLD After Bariatric Surgery. Int. J. Mol. Sci..

[71] Cremades M., Talavera-Urquijo E., Beisani M., Pappa S., Jordà M., Tarascó J., Moreno P., Caballero A., Martínez-López E., Pellitero S. (2024). Transcriptional and Epigenetic Changes after Dietary and Surgical Weight Loss Interventions in an Animal Model of Obesity. Int. J. Obes..

[72] Sangiao-Alvarellos S., Theofilatos K., Barwari T., Gutmann C., Takov K., Singh B., Juiz-Valiña P., Varela-Rodríguez B.M., Outeiriño-Blanco E., Duregotti E. (2020). Metabolic Recovery after Weight Loss Surgery Is Reflected in Serum microRNAs. BMJ Open Diabetes Res. Care.

[73] Pan Y., Hui X., Hoo R.L.C., Ye D., Chan C.Y.C., Feng T., Wang Y., Lam K.S.L., Xu A. (2019). Adipocyte-Secreted Exosomal microRNA-34a Inhibits M2 Macrophage Polarization to Promote Obesity-Induced Adipose Inflammation. J. Clin. Investig..

[74] Cornejo P.-J., Vergoni B., Ohanna M., Angot B., Gonzalez T., Jager J., Tanti J.-F., Cormont M. (2022). The Stress-Responsive microRNA-34a Alters Insulin Signaling and Actions in Adipocytes through Induction of the Tyrosine Phosphatase PTP1B. Cells.

[75] Fraszczyk E., Luijten M., Spijkerman A.M.W., Snieder H., Wackers P.F.K., Bloks V.W., Nicoletti C.F., Nonino C.B., Crujeiras A.B., Buurman W.A. (2020). The effects of bariatric surgery on clinical profile, DNA methylation, and ageing in severely obese patients. Clin. Epigenet..

[76] Yadav J., Liang T., Qin T., Nathan N., Schwenger K.J.P., Pickel L., Xie L., Lei H., Winer D.A., Maughan H. (2023). Gut microbiome modified by bariatric surgery improves insulin sensitivity and correlates with increased brown fat activity and energy expenditure. Cell Rep. Med..

[77] Azari H., George M., Albracht-Schulte K. (2024). Gut Microbiota-microRNA Interactions and Obesity Pathophysiology: A Systematic Review of Integrated Studies. Int. J. Mol. Sci..

[78] Kato S., Waki H. (2025). Decoding the adipocyte epigenome: Differentiation, metabolic memory, and obesity. J. Obes. Metab. Syndr..

[79] Long Y., Mao C., Liu S., Tao Y., Xiao D. (2024). Epigenetic modifications in obesity-associated diseases. MedComm.

[80] Zahoor I., Mir G.J., Lone N.A., Ashraf N.U. (2025). Crosstalk between Epigenetics and Autophagy in Metabolic Dysfunction-Associated Steatotic Liver Disease. J. Obes. Metab. Syndr..

[81] Dong T.S., Katzka W., Yang J.C., Chang C., Arias-Jayo N., Lagishetty V., Balioukova A., Chen Y., Dutson E., Li Z. (2023). Microbial changes from bariatric surgery alters glucose-dependent insulinotropic polypeptide and prevents fatty liver disease. Gut Microbes.

[82] Yuan X., Tan Y., Bajinka O., Jammeh M.L., Dukureh A., Obiegbusi C.N., Abdelhalim K.A., Mohanad M. (2024). The connection between epigenetics and gut microbiota-current perspective. Cell Biochem. Funct..

[83] Wu Y.-L., Lin Z.-J., Li C.-C., Lin X., Shan S.-K., Guo B., Zheng M.-H., Li F., Yuan L.-Q., Li Z.-H. (2023). Epigenetic regulation in metabolic diseases: Mechanisms and advances in clinical study. Signal Transduct. Target. Ther..

[84] Blasco-Baque V., Coupé B., Fabre A., Handgraaf S., Gourdy P., Arnal J.-F., Courtney M., Schuster-Klein C., Guardiola B., Tercé F. (2017). Associations between hepatic miRNA expression, liver triacylglycerols and gut microbiota during metabolic adaptation to high-fat diet in mice. Diabetologia.

[85] Georgiou K., Belev N.A., Koutouratsas T., Katifelis H., Gazouli M. (2022). Gut microbiome: Linking together obesity, bariatric surgery and associated clinical outcomes under a single focus. World J. Gastrointest. Pathophysiol..

[86] Wågen Hauge J., Borgeraas H., Birkeland K.I., Johnson L.K., Hertel J.K., Hagen M., Gulseth H.L., Lindberg M., Lorentzen J., Seip B. (2025). Effect of gastric bypass versus sleeve gastrectomy on the remission of type 2 diabetes, weight loss, and cardiovascular risk factors at 5 years (Oseberg): Secondary outcomes of a single-centre, triple-blind, randomised controlled trial. Lancet Diabetes Endocrinol..

[87] Trøseid M., Hov J.R., Nestvold T.K., Thoresen H., Berge R.K., Svardal A., Lappegård K.T. (2016). Major Increase in Microbiota-Dependent Proatherogenic Metabolite TMAO One Year After Bariatric Surgery. Metab. Syndr. Relat. Disord..

[88] Chakaroun R.M., Olsson L.M., Bäckhed F. (2023). The potential of tailoring the gut microbiome to prevent and treat cardiometabolic disease. Nat. Rev. Cardiol..

[89] Leishman D., Ikramuddin S. (2023). What happens to adipose tissue after obesity surgery. BJSA.

[90] Wang H. (2022). Role of EZH2 in adipogenesis and obesity: Current state of the art and implications—A review. Medicine.

[91] Latteri S., Sofia M., Puleo S., Di Vincenzo A., Cinti S., Castorina S. (2023). Mechanisms linking bariatric surgery to adipose tissue, glucose metabolism, fatty liver disease and gut microbiota. Langenbecks Arch. Surg..

[92] Tuomi K., Logomarsino J.V. (2016). Bacterial Lipopolysaccharide, Lipopolysaccharide-Binding Protein, and Other Inflammatory Markers in Obesity and After Bariatric Surgery. Metab. Syndr. Relat. Disord..

[93] Swierz M.J., Storman D., Staskiewicz W., Gorecka M., Jasinska K.W., Swierz A.M., Tobola P., Skuza A., Bala M.M. (2020). Efficacy of probiotics in patients with morbid obesity undergoing bariatric surgery: A systematic review and meta-analysis. Surg. Obes. Relat. Dis..

[94] Daghmouri M.A., Chaouch M.A., Yang W., Akremi S., Jaoua H., Fadhel K.B., Gouader A., Reissfelder C., Elhadedy H., Rahbari N. (2022). Probiotics in bariatric surgery ensure greater lipids and glycemic profile with no effect on anthropometric measurements and inflammatory markers: A systematic review and meta-analysis of RCT. Surg. Open Dig. Adv..

[95] Ezzi M.Y. (2026). Probiotics After Metabolic and Bariatric Surgery: A Systematic Review and Meta-Analysis of Randomized Controlled Trials. Metabolites.

[96] Sherf-Dagan S., Zelber-Sagi S., Zilberman-Schapira G., Webb M., Buch A., Keidar A., Raziel A., Sakran N., Goitein D., Goldenberg N. (2018). Probiotics administration following sleeve gastrectomy surgery: A randomized double-blind trial. Int. J. Obes..

[97] Fernandes R., Beserra B.T.S., Mocellin M.C., Kuntz M.G.F., da Rosa J.S., de Miranda R.C.D., Schreiber C.S.O., Fröde T.S., Nunes E.A., Trindade E.B.S.M. (2016). Effects of Prebiotic and Synbiotic Supplementation on Inflammatory Markers and Anthropometric Indices After Roux-en-Y Gastric Bypass: A Randomized, Triple-blind, Placebo-controlled Pilot Study. J. Clin. Gastroenterol..

[98] Lahtinen P., Juuti A., Luostarinen M., Niskanen L., Liukkonen T., Tillonen J., Kössi J., Ilvesmäki V., Viljakka M., Satokari R. (2022). Effectiveness of fecal microbiota transplantation for weight loss in patients with obesity undergoing bariatric surgery: A randomized clinical trial. JAMA Netw. Open.

[99] Guo M., Li J., Zhang L., Chen C., Wei Y., Shen Z.-A. (2025). Effects of oral supplementation of probiotics on body weight and visceral fat in obese patients: A meta-analysis and systematic review. Sci. Rep..

[100] Ejtahed H.-S., Angoorani P., Soroush A.-R., Atlasi R., Hasani-Ranjbar S., Mortazavian A.M., Larijani B. (2019). Probiotics supplementation for the obesity management; A systematic review of animal studies and clinical trials. J. Funct. Foods.

[101] Mokhtari Z., Karbaschian Z., Pazouki A., Kabir A., Hedayati M., Mirmiran P., Hekmatdoost A. (2019). The Effects of Probiotic Supplements on Blood Markers of Endotoxin and Lipid Peroxidation in Patients Undergoing Gastric Bypass Surgery; a Randomized, Double-Blind, Placebo-Controlled, Clinical Trial with 13 Months Follow-Up. Obes. Surg..

[102] Ramos M.R.Z., de Oliveira Carlos L., Wagner N.R.F., Felicidade I., da Cruz M.R., Taconeli C.A., Fernandes R., Filho A.J.B., Campos A.C.L. (2021). Effects of Lactobacillus acidophilus NCFM and Bifidobacterium lactis Bi-07 Supplementation on Nutritional and Metabolic Parameters in the Early Postoperative Period after Roux-en-Y Gastric Bypass: A Randomized, Double-Blind, Placebo-Controlled Trial. Obes. Surg..

[103] Lo Menzo E., Cappellani A., Zanghì A., Di Vita M., Berretta M., Szomstein S. (2014). Nutritional implications of obesity: Before and after bariatric surgery. Bariatr. Surg. Pract. Patient Care.

[104] Rubas N.C., Torres A., Maunakea A.K. (2025). The gut microbiome and epigenomic reprogramming: Mechanisms, interactions, and implications for human health and disease. Int. J. Mol. Sci..

[105] Duan D., Wang M., Han J., Li M., Wang Z., Zhou S., Xin W., Li X. (2024). Advances in multi-omics integrated analysis methods based on the gut microbiome and their applications. Front. Microbiol..

[106] Kostka A., Gruszecka-Kosowska A., Ruiz-Rodríguez A., Aguilera M. (2024). Multi-omics approach in gut and environmental microbiota research under the One Health concept. EFSA J..

[107] Wang Q., Wang K., Wu W., Giannoulatou E., Ho J.W.K., Li L. (2019). Host and microbiome multi-omics integration: Applications and methodologies. Biophys. Rev..

[108] Jastreboff A.M., Aronne L.J., Ahmad N.N., Wharton S., Connery L., Alves B., Kiyosue A., Zhang S., Liu B., Bunck M.C. (2022). Tirzepatide Once Weekly for the Treatment of Obesity. N. Engl. J. Med..

[109] Jastreboff A.M., Kaplan L.M., Frías J.P., Wu Q., Du Y., Gurbuz S., Coskun T., Haupt A., Milicevic Z., Hartman M.L. (2023). Triple–Hormone-Receptor Agonist Retatrutide for Obesity—A Phase 2 Trial. N. Engl. J. Med..

[110] Debédat J., Clément K., Aron-Wisnewsky J. (2019). Gut microbiota dysbiosis in human obesity: Impact of bariatric surgery. Curr. Obes. Rep..

[111] Morales-Marroquin E., Hanson B., Greathouse L., de la Cruz-Munoz N., Messiah S.E. (2020). Comparison of methodological approaches to human gut microbiota changes in response to metabolic and bariatric surgery: A systematic review. Obes. Rev..

[112] Fusco W., Lorenzo M.B., Cintoni M., Porcari S., Rinninella E., Kaitsas F., Lener E., Mele M.C., Gasbarrini A., Collado M.C. (2023). Short-Chain Fatty-Acid-Producing Bacteria: Key Components of the Human Gut Microbiota. Nutrients.

[113] Reva K., Laranjinha J., Rocha B.S. (2023). Epigenetic Modifications Induced by the Gut Microbiota May Result from What We Eat: Should We Talk about Precision Diet in Health and Disease?. Metabolites.

